# A telemedicine-based approach with real-time transmission of blood glucose data improves metabolic control in insulin-treated diabetes: the DIAMONDS randomized clinical trial

**DOI:** 10.1007/s40618-022-01802-w

**Published:** 2022-04-27

**Authors:** S. Di Molfetta, P. Patruno, S. Cormio, A. Cignarelli, R. Paleari, A. Mosca, O. Lamacchia, S. De Cosmo, M. Massa, A. Natalicchio, S. Perrini, L. Laviola, F. Giorgino

**Affiliations:** 1 Section of Internal Medicine, Endocrinology, Andrology and Metabolic Diseases, Department of Emergency and Organ Transplantation, University of Bari Aldo Moro, Bari, Italy; 2 Section of Endocrinology, Department of Medical and Surgical Sciences, University of Foggia, Foggia, Italy; 3 Section of Internal Medicine, Department of Medical Sciences, IRCCS “Casa Sollievo della Sofferenza”, San Giovanni Rotondo, Foggia, Italy; 4 Department of Physiopathology and Transplantation, Center for Metrological Traceability in Laboratory Medicine (CIRME), University of Milan, Milan, Italy

**Keywords:** Telemedicine, SMBG, Glucoonline, Diabetes mellitus, Randomized controlled trial

## Abstract

**Purpose:**

To evaluate if a web-based telemedicine system (the Glucoonline^®^ system) is effective to improve glucose control in insulin-treated patients with type 1 and type 2 diabetes, as compared to standard of care.

**Methods:**

This was a prospective, randomized, controlled trial, carried out at three tertiary referral centers for diabetes in Italy. Adults with insulin-treated type 1 and type 2 diabetes, inadequate glycemic control, and no severe diabetes-related complications and/or comorbidities were eligible for this study. Patients were randomized to either perform telemedicine-assisted (Group A) or standard (Group B) self-monitoring blood glucose (SMBG) for 6 months. In Group A, patients received prompt feedback about their blood glucose levels and therapy suggestions from the study staff via phone/SMS, when appropriate. In Group B, patients had no remote assistance from the study staff between planned visits.

**Results:**

123 patients were included in the final analysis. After 6 months, patients achieved a significant reduction in HbA1c in Group A (−0.38%, *p* < 0.05) but not in Group B (+ 0.08%, *p* = 0.53). A significant difference in the percentage of patients with HbA1c < 7% between Group A and Group B was found after 3 months (28.6% vs 11.1%, *p* = 0.02). Also, fewer patients (*p* < 0.05) with HbA1c > 8.5% were found in Group A vs Group B, respectively, after both 3 months (14.3% vs 35.2%) and 6 months (21.8% vs 42.9%).

**Conclusions:**

The use of the Glucoonline™ system resulted in improved metabolic control. Telemedicine services have potential to support diabetes self-management and provide the patients with remote, prompt assistance using affordable technological equipment.

**Trial registration** This study was registered at clinicaltrials.gov (NCT01804803) on March 5, 2013.

## Introduction

Self-monitoring of blood glucose (SMBG) is a cornerstone of diabetes management, as it may help detect the effects of glucose-lowering therapies and patient lifestyle on individual blood glucose (BG) levels [1–3]. International guidelines recommend individualized SMBG frequency based on multiple factors, including type of diabetes, treatment regimen (i.e., lifestyle intervention, non-insulin oral and injectable agents, or insulin), quality of glycemic control, risk of hypoglycemia, and patient's willingness to self-test [4, 5]. Accordingly, most patients using intensive insulin therapy (multiple daily injections or insulin pump therapy) are required to check their BG at least 3–4 times daily [6].

However, to fully exploit SMBG and achieve glucose targets, SMBG should be performed in a structured manner (e.g., before meals and snacks, at bedtime, occasionally postprandially, before exercise, when hypoglycemia is suspected, before driving, etc.), and the results need to be accurately interpreted and used to guide adjustments in medications, especially insulin, and to modify food intake and/or physical activity [7, 8]. Nevertheless, a substantial proportion of patients who report checking their BG at least once daily also report taking no action when results show high or low values [9].

We have developed a novel system [Glucoonline^®^, 2011], comprised of a smartphone-connectable glucose meter, a software-implemented smartphone for real-time BG data transmission, and a Decision Support Software (DSS)-assisted remote server performing comprehensive data analysis, and providing prompt feedback to the patient and the health care provider (HCP) through predefined algorithms. Also, the system could help to implement multiple aspects of diabetes management, including assessment and enhancement of patients’ adherence to the recommended SMBG scheme, HCP’s evaluation of overall glucose control, detection of actual/impending hyper- and/or hypoglycemic events, and promptness of intervention in case of emergency situations. The feasibility of such a system for clinical use was preliminarly evaluated over a 3-month period in a pilot study enrolling 10 individuals with type 1 diabetes on multiple daily  insulin injections (data on file).

The main aim of this study was to investigate if the web-based Glucoonline^®^ system is effective in improving overall glucose control, determined by measuring HbA1c levels, in insulin-treated patients with type 1 and type 2 diabetes, as compared to standard of care.

## Methods

### Trial design

This was a prospective, randomized, controlled trial, recruiting patients from 3 sites (site No. 1, located in Bari; site No. 2, located in Foggia; site No. 3, located in S. Giovanni Rotondo) in Italy. Patients matching the eligibility criteria and willing to participate with informed consent were randomized to perform either telemedicine-assisted (Group A) or standard (Group B) SMBG for 6 months. The study was submitted to local institutional ethics committees (protocol no. 925, approved on August 2, 2012, by the Institutional Ethics Committee of the University Hospital Policlinico Consorziale, Bari, Italy, as the coordinating center) and carried out in adherence to Good Clinical Practice, ICH Harmonized Tripartite Guidelines for Good Clinical Practice and Declaration of Helsinki. The study was registered at clinicaltrials.gov (unique identifier: NCT01804803).

### Participants

Patients had to meet the following inclusion criteria: males and females; age 18–70 years; insulin-treated diabetes (both type 1 and type 2 diabetes treated with at least 3 injections/day regardless of type of insulin); diagnosis of diabetes from at least 1 year; inadequate glycemic control (HbA1c ranging from 7.0% to 10.0%; local measurements within the last 6 weeks); ability and willingness to carry out SMBG; informed consent. Major exclusion criteria were: established and impending complications of diabetes, such as proliferative retinopathy or maculopathy (with significant loss of visual function), severe renal failure (eGFR < 30 ml/min/m^2^), severe neuropathy (autonomic dysfunction, peripheral neuropathy, gastroparesis); clinically significant, active (over the past 12 months) disease of the cardiovascular, gastrointestinal, neurological, genito-urinary, or hematological systems; severe uncontrolled hypertension (systolic blood pressure > 180 mmHg; diastolic blood pressure > 100 mmHg); diagnosis of active neoplasia within the last 5 years (or history of chemotherapy/radiation-treated malignancy within 5 years prior to study procedure, except for lymphoma); pregnancy or intention to become pregnant during the study; any other concomitant medical or psychological condition which, in the judgment of the investigator, could make the patient unsuitable for study participation.

### Procedures

All patients were instructed to perform SMBG according to the national Italian guidelines (4 SMBG tests per day or more in case of metabolic derangement) [10] and adjust insulin doses based on measured glucose readings. The recommended SMBG scheme included testing in the fasting state, before and 2 h after breakfast and/or lunch, and/or dinner, and 3–5 h after lunch or dinner (absorptive phase).

**Fig.1:**
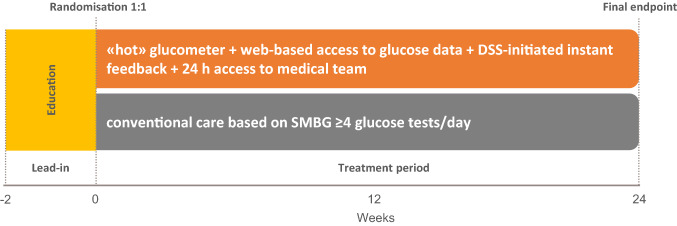
Study design. DSS, decision support software; SMBG, self-monitoring of blood glucose. Glucometer is defined as «hot» since it was associated with an external device for real-time transmission of glucose data

Randomization was carried out in a 1:1 manner using a web-based application, and stratified by age, sex, type of diabetes, and HbA1c level (≤ 8.5%, > 8.5%). The study design is illustrated in Fig. 1. Patients in Group A were given a smartphone-connectable meter (OneTouchPro Verio, Lifescan, modified for USB cable connection to smartphone) and a software-implemented smartphone for real-time data transmission. Wireless connectivity between the smartphone and the remote server was enabled, ensuring a bidirectional data flow. Also, a web-based electronic CRF (Glucoonline™ eCRF) was created, allowing for multiple assessments: (i) appropriateness of SMBG frequency; (ii) overall glucose control quality; (iii) graphical visualization of BG values according to meals and time of the day; (iv) absolute number and percentage of BG values < 70 mg/dl, < 50 mg/dl and < 30 mg/dl; (v) absolute number and percentage of BG values > 180 mg/dl, > 250 mg/dl, and > 350 mg/dl; (vi) the low blood glucose index (LBGI), high blood glucose index (HBGI), and Average Daily Risk Range (ADRR) developed by Kovatchev et al. [11–13]. Patients in Group B were given a regular glucose meter (OneTouchPro Verio, Lifescan) and were asked to report their glucose levels on a paper diary.

At the first visit (V1), patients in Group A underwent an additional educational session to learn how to use the meter and transmit BG data, log into their personal Glucoonline™ eCRF and access meaningful information about their glucose control, interpret their glucose patterns, and refer themselves to the medical staff irrespective of the planned study visits when needed. After V1, all patients had two follow-up visits with a 3-month interval (V2 and V3). In the intervention arm, investigators regularly checked the Glucoonline™ eCRF throughout the study period; also, they received an alert by the DSS-supported server every time an individual patient (i) was performing SMBG sub-optimally (e.g., infrequent or temporally inadequate testing), (ii) had displayed BG values beyond thresholds set for hypoglycemia/hyperglycemia, and (iii) had experienced recurrent hypoglycemia or sustained hyperglycemia. Under these conditions, irrespective of the planned study visits, investigators could make prompt interventions, including patient counseling via phone/SMS, or arrange for a medical visit. Specific interventions could be implemented if patients had SMBG values < 40 mg/dl. In the control arm (Group B), patients received no feedback about their BG levels from the study staff between planned visits, nor instructions on how to modify their therapy. Also, they had no remote assistance in case of emergency situations. At V2 and V3, the medical personnel could make insulin dose and other therapy adjustments according to their clinical judgement. At each study visit, patients underwent comprehensive physical examination and had blood samples collected for HbA1c, fasting glucose (FPG), creatinine, and lipid profile measurements. The HbA1c measurements were performed by HPLC. The Bio-Rad Variant II system (Bio-Rad Laboratories, Segrate, Italy) was used in sites No. 1 and 3 and the Tosoh G8 (Tosoh Bioscience, Torino, Italy) in site No. 2. The analytical performances of the local laboratories during the whole study were evaluated based on their internal quality control (IQC) results and from the participation to the External Quality Assessment (EQAS) programs.

### Outcomes

The primary endpoint was change in HbA1c from V1 assessed at V3 (6 months). Also, differences in the following secondary endpoints were evaluated: change in HbA1c from V1 assessed at V2 (3 months), proportion of patients with HbA1c > 8.5%, < 7.0% or < 6.5%; frequency of BG testing; conformity with recommended SMBG scheme; fasting BG (FBG); postprandial BG (PPBG); post/preprandial BG excursion (PPBGE); SMBG-derived indices of overall glycemic control and variability; frequency of hypoglycemic episodes. Conformity with recommended SMBG scheme was defined as the ratio of days with appropriate BG testing to the total days and expressed as percentage of days.

### Sample size

The sample size was calculated based on a previous study [14] evaluating the efficacy of a telemonitoring intervention, coupled with active medication management, in reducing HbA1c, as compared with the control group. Accordingly, it was established that at least 88 patients (44 per group) were needed to find a difference with a power of 80% and significance level of 5%.

### Statistical analysis

Continuous variables are expressed as mean ± standard deviation and categorical variables as count (percentage). A two-tailed paired Student's t-test and a chi-square test were run to test for differences in continuous and categorical variables, respectively. An ANOVA test was run to check differences in SMBG data at different time points. The Shapiro–Wilk test was used to assess for data normality. All statistical analyses and data processing were performed using SPSS^®^ Software (version 19, IBM). A two-sided *p*-value < 0.05 was regarded as statistically significant.

## Results

**Fig. 2:**
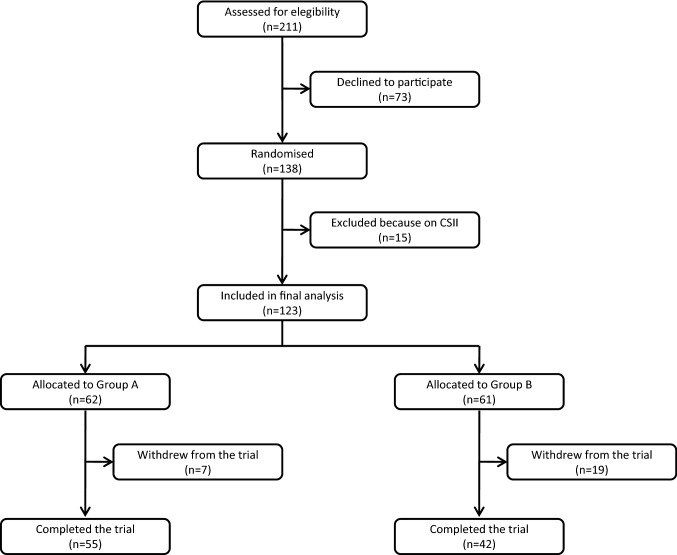
Diagram illustrating patient flow from assessment to completion of the study protocol

The study started in September 2013 and ended in November 2017. Procedures and devices were kept identical throughout this time frame. The total number of patients evaluated in the study was 211, of which 123 were included in the final analysis and 97 completed the 6-month follow-up period (Fig. 2). Most common reasons for declining participation in the trial were poor compliance with self-testing of blood glucose and/or unwillingness to be engaged in an intensive monitoring program involving use of smartphone and device. Patients randomized to either Group A or Group B did not show significant clinical or biochemical differences at baseline (Table 1).

**Table 1:** Baseline characteristics of study participants.

Variable	Group A	Group B	*p*-value
*N*	62	61	
Site			0.996
No. 1, *n* (%)	30 (48.4)	29 (47.5)	
No. 2, *n* (%)	9 (14.5)	9 (14.8)	
No. 3, *n* (%)	23 (37.1)	23 (37.7)	
Males, *n* (%)	33 (53.2)	34 (55.7)	0.921
Age, years	47.15 ± 14.54	45.21 ± 14.76	0.466
Diabetes duration, years	32.85 ± 15.36	30.63 ± 15.41)	0.432
Diabetes, type 2, *n* (%)	32 (51.6)	20 (32.8)	0.054
HbA1c, %	8.00 ± 0.97	8.28 ± 1.25	0.175
BMI, kg/m^2^	27.23 ± 5.91	26.54 ± 5.07	0.489
Waist, cm	94.88 ± 15.01	92.09 ± 14.45	0.325
Systolic blood pressure, mmHg	122.95 ± 17.18	123.67 ± 18.22	0.824
Diastolic blood pressure, mmHg	75.25 ± 7.72	76.17 ± 8.99	0.546

Continuous variables are expressed as mean ± standard deviation.

*BMI*, body mass index; *HbA1c*, glycated hemoglobin

### Primary endpoint

**Fig. 3:**
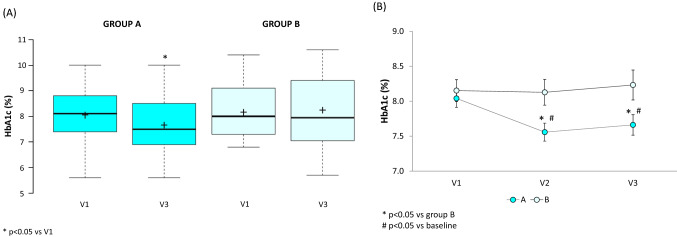
Local laboratory-measured HbA1c as assessed at study end versus V1 **A** and at each time point **B** in Group A and Group B

At V3, patients achieved a significant reduction in HbA1c level compared to baseline in Group A (0.38% versus V1, *p* < 0.05, paired *t*-test) but not in Group B (+ 0.08% versus V1, *p* = 0.5345, paired *t*-test) (Fig. 3).

### Secondary endpoints

**Fig. 4:**
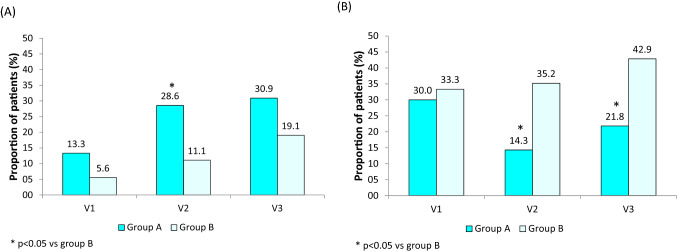
Proportion of patients achieving a HbA1c level of less than 7% **A** and above 8.5% **B** at each time point in Group A and Group B

A higher percentage of patients with HbA1c < 7% was observed in Group A than in Group B at V2 (*p* = 0.022, *χ*^2^ test). Also, fewer patients with HbA1c > 8.5% were found at both V2 (*p* = 0.011, *χ*^2^ test) and V3 (*p* = 0.026, *χ*^2^ test) in Group A versus Group B (Fig. 4).

In patients randomized to Group A, SMBG-derived indices were analyzed for the following periods: 14 days following V1 (T1), 14 days preceding V2 (T2), and 14 days preceding V3 (T3). No significant differences were observed in regard to mean BG, coefficient of variation, percentage of data in the 70–180 mg/dl range, percentage of data below the 70–180 mg/dl range, percentage of data above the 70–180 mg/dl range, fasting BG, postprandial BG, post/pre-breakfast BG difference, post/pre-lunch BG difference, post/pre-dinner BG difference, number of BG values per day and conformity to recommended SMBG scheme (Table 2). In group B, very few BG readings were available on paper diaries at *T*1; at *T*2 and *T*3, the testing frequency was 2.5 and 2.4 tests per day, respectively.

**Table 2:** SMBG-derived indices analyzed for (i) 14 days following V1 (T1), (ii) 14 days preceding V2 (T2), and (iii) 14 days preceding V3 (T3).

Variable	*T*1	*T*2	*T*3	*p*-value
MBG, mg/dl	152.3 ± 26.3	152.7 ± 27.4	151.3 ± 23.6	0.923194
CV, %	39.0 ± 13.2	37.4 ± 10.3	38.9 ± 13.1	0.351657
BG < 70 mg/dl, %	6.6 ± 8.7	6.2 ± 6.1	6.2 ± 8.7	0.914028
70 ≤ BG ≤ 180 mg/dl, %	67.1 ± 20.0	66.0 ± 20.9	66.7 ± 16.5	0.904956
BG ≥ 180 mg/dl, %	26.3 ± 18.2	27.8 ± 19.1	27.1 ± 14.7	0.852430
FBG, mg/dl	147.6 ± 34.1	151.3 ± 37.1	153.3 ± 29.6	0.568204
PPBG, mg/dl	158.1 ± 37.3	153.2 ± 44.3	156.5 ± 51.0	0.764194
Breakfast PPBGE, mg/dl	37.7 ± 43.3	18.4 ± 54.1	6.1 ± 56.7	0.337702
Lunch PPBGE, mg/dl	19.0 ± 51.8	9.6 ± 34.2	-9.7 ± 44.0	0.123968
Dinner PPBGE, mg/dl	3.8 ± 48.4	14.1 ± 79.1	14.1 ± 55.5	0.701380
BG/day, *n*	3.1 ± 1.3	3.1 ± 1.3	3.0 ± 1.4	0.887088
Conformity, %	46.2 ± 35.3	46.5 ± 38.6	44.9 ± 38.2	0.951261

Data are expressed as mean ± standard deviation.

*BG*, blood glucose; *CV*, coefficient of variation; *FBG*, fasting blood glucose; *MBG*, mean blood glucose; *PPBG*, postprandial blood glucose; *PPBGE*, postprandial blood glucose excursion

In addition, no significant changes were observed in terms of body weight or waist circumference changes at V3 versus baseline in either Group A or Group B patients (data not shown).

## Discussion

### Principal findings

In patients with insulin-treated type 1 and type 2 diabetes the use of the Glucoonline™ system resulted in a 0.38% decrease in HbA1c from baseline and a higher proportion of patients achieving a HbA1c target level of less than 7%, as compared with standard of care. Of note, benefits were found already after a 3-month observation period and were maintained until study end. Thus, this telemonitoring system paired to a web-based DSS resulted in a both statistically and clinically significant improvement in metabolic control. In the intervention group, accurate BG reporting and enhanced data analysis through the web-based eCRF, together with the possibility to provide the patients with timely feedback for hyperglycemia management, may have resulted in better metabolic control, even though we could not document a clear improvement in either SMBG testing frequency or daily profiles. On the other hand, the Glucoonline™ system did not promote a reduction of body weight or waist circumference.

### Strengths and limitations of study

There is growing evidence to support telemedicine as a valuable intervention to improve glucose control with reduction of HbA1c levels and diabetes-related adverse outcomes in patients with both type 1 and type 2 diabetes [15]. However, studies dealing with telemedicine facilities in diabetes management to date suffer from important and underappreciated pitfalls (e.g., small sample size, lack of controls, poor study design, lack of demonstration of a long-term benefit, etc.). Our study had an experimental design with active control group and a 6-month follow-up period; also, an adequate retention rate (> 80%) was achieved at study end.

Nevertheless, this study has some limitations. First, no SMBG data were collected prior to study intervention, possibly preventing detection of potential intervention-related improvements in daily self-monitoring of glucose levels. Indeed, SMBG data indicate a relatively good glucose control since T1 with a percentage of readings in the glucose range 70–180 mg/dl of 67%. Second, we could not use central laboratory-data analysis. However, the intra-lab imprecision for HbA1c measured at the local laboratories, expressed as coefficient of variation, was between 1.1 and 5.0% in the normal HbA1c range (i.e., HbA1c level ranging from 5.1 to 5.7%), and between 0.8 and 3.8% in the elevated HbA1c range (i.e., HbA1c level ranging from 9.9 to 10.7%). Also, in years 2015–2017, the relative biases respect to the target values in the EQAS exercises were between 0.4 and 2.8% for site No. 1, and between 0.5 and 1.1% for site No. 2, thus proving that the methods were sufficiently aligned. Third, it was not possible to retrieve adequate information on SMBG-driven instant messaging, physician-to-participant contact frequency, and change in total insulin daily dose, which would all give further consistency to our results and provide potential additional interpretations of the findings. Fourth, the proportion of subjects with type 2 diabetes was numerically higher in group A as compared with group B, even though this difference did not reach statistical significance. As patients with type 2 diabetes may achieve larger HbA1c reductions with telemedicine-based interventions than patients with type 1 diabetes [15, 16], this unbalance may have affected the results of the trial. Fifth, clinicians were not blinded to group allocation, thus a bias in the delivery of the intervention cannot be excluded.

### Comparison with other studies

Two recent meta-analyses of randomized controlled trials have shown a mean difference of 0.48% and 0.37% for change in HbA1c with telemedicine interventions in patients with type 1 and type 2 diabetes, respectively [15, 16]. These findings are in line with our results, since HbA1c declined in the Glucoonline™-assisted intervention group but not in the control group, with a mean 0.46% difference in HbA1c between groups at study end. Evidence from the existing literature also supports the effectiveness of digital self-management interventions after 3–6 months [16–18], with digital health education programs requiring a longer duration of the intervention period to obtain a significant reduction in HbA1c [19].

Regarding changes in body weight and/or composition, indeed previous studies found conflicting results, showing either some improvement [20–23] or no difference [24, 25] with telediabetes services as compared to usual care. In our study, no change in body weight or waist circumference occurred in the interventions group; however, the Glucoonline™ system was not implemented to also track body weight measures and send support messages for changes in physical activity or diet, or for emphasizing potential weight loss.

### Clinical and policy implications

With the advances in technology and increased use of web-based applications, multiple telemedicine options are available to help the patients manage their diabetes and achieve improved treatment outcomes. Telemedicine may enable an active interaction between people with diabetes and healthcare professionals by encouraging adequate SMBG testing frequencies and modalities, making SMBG results available for analyses to assess the quality of glucose control and providing the patient with appropriate SMBG-driven therapy adjustments. Telemedicine also could allow to check updated SMBG data to detect emergency situations (e.g., severe hypoglycemia, persistent hyperglycemia) and assist the patient with timely interventions. Accordingly, telemedicine services are appropriate to support frail patients with a high risk of diabetes-related adverse complications and events [26, 27]. Furthermore, the recent outbreak of SARS-CoV-2 pandemic has legitimized telemedicine as a valuable solution to monitor and assist a large number of patients at their homes remotely, thus relieving patient flow to the outpatient clinics [28, 29]. Our study provides additional evidence of the benefits of telemedicine strategies in patients with insulin-treated diabetes and demonstrates for the first time that the combination of a smartphone-based BG transmission and a DSS-assisted feedback technology results in clinically relevant improvement in metabolic control.

## Conclusion

The use of the web-based Glucoonline™ system for 6 months resulted in improved glucose control in patients with insulin-treated type 1 and type 2 diabetes, as compared to standard of care. Telemedicine services have great potential to support diabetes self-management and provide remote, prompt assistance with affordable technological equipment.

## Data Availability

The dataset of this study is not publicly available but is available from the corresponding author on reasonable request.
